# A Study on the Gamma Radiation Protection Effectiveness of Nano/Micro-MgO-Reinforced Novel Silicon Rubber for Medical Applications

**DOI:** 10.3390/polym14142867

**Published:** 2022-07-14

**Authors:** M. I. Sayyed, Hanan Al-Ghamdi, Aljawhara H. Almuqrin, Sabina Yasmin, Mohamed Elsafi

**Affiliations:** 1 Department of Physics, Faculty of Science, Isra University, Amman 11622, Jordan; 2 Department of Physics, College of Science, Princess Nourah Bint Abdulrahman University, P.O.Box 84428, Riyadh 11671, Saudi Arabia; hmalghmdi@pnu.edu.sa (H.A.-G.); ahalmoqren@pnu.edu.sa (A.H.A.); 3 Department of Physics, Chittagong University of Engineering and Technology, Chattogram 4349, Bangladesh; sabinayasmin309@gmail.com; 4 Physics Department, Faculty of Science, Alexandria University, Alexandria 21511, Egypt; mohamedelsafi68@gmail.com

**Keywords:** radiation protection efficiency, HPGe detector, gamma radiation, EDX analysis

## Abstract

In this work, we examined novel polymer composites for use in radiation protection applications. These prepared polymers are non-toxic compared with lead and show potential to be used as protective gear in different medical applications where low-energy photons are utilized. We prepared silicon rubber (SR) with different concentrations of micro- and nano-sized MgO. We used a HPGe detector to measure radiation attenuation factors at different photon energies, ranging from 59.6 to 1333 keV. We reported the effect of particle size on the attenuation parameters and found that the linear attenuation factors for SR with nano-MgO were higher than for SR with micro-MgO. The mean free path (MFP) for pure SR and SR with micro- and nano-sized MgO were determined, and we found that silicon rubber with MgO (both micro- and nano-sized) has a lower MFP than pure SR. The linear attenuation coefficient results show the importance of using SR with high MgO content for low-energy radiation protection applications. Moreover, the half-value layer (HVL) results demonstrate that we need a certain thickness of SR with nano-MgO to effectively reduce the intensity of the low-energy photons.

## 1. Introduction

Over the past century, ionizing radiation has been widely used in many aspects of society, including radiation medicine, the nuclear power industry, aerospace exploration industries, and nuclear research laboratories. The safety of workers, patients, and equipment has become a fundamental issue due to the hazards of working with radiation. High-energy radiation has strong penetrability and thus has hazardous effects on both equipment and the human body. Radiation shielding materials must be used to reduce the hazardous effects of radiation [1,2,3].

Lead and concrete are the conventional materials used for this purpose. As these materials have poor mechanical properties and are opaque, they cannot be used to protect the eyes and face from hazardous radiation. Moreover, they can be toxic. Novel radiation shielding materials must fit certain criteria, such as having low weight, high mechanical strength, flexibility, movability, and a high absorption capability against gamma photons, to be considered an adequate replacement for the traditionally used materials [4,5,6,7].

Recently, polymer composites have introduced a new generation of lightweight hybrid materials for radiation shielding. Polymer composites offer wide utilization in different industrial, medical, and technological fields [8,9,10]. In the last few years, several research labs have focused on preparing polymers with appropriate fillers to overcome the drawbacks of traditional shielding materials. There is little literature on the use of polymer composites in radiation shielding. Almurayshid et al., researched the possibility of several high-density polyethylene (HDPE) polymer composites doped in molybdenum (Mo), molybdenum carbide (MoC), tungsten (W), and tungsten carbide (WC), to be used against kilovoltage X-ray shielding. Considering the obtained value of mass attenuation coefficient and equivalent atomic number, the tungsten (W) and tungsten carbide (WC) composites showed the best shielding capabilities among the studied samples [11].

Turhan et al., studied polymer composites with an added 25, 50, 75, and 100% hematite for fortification against gamma radiation. They assessed the transmission factor, linear attenuation coefficients, electron density, and other related parameters of prepared polymer composites. The mass attenuation coefficients of 100% hematite-doped polymer composites within the energy range of 59.5–1408.0 keV showed a better gamma radiation shielding efficiency than other composites [12]. Gilys et al., studied a newly established lead-free multi-layered structure grounded in silicone composite layers, containing tin, CeO_2_, WO_3_, and bismuth additives, and examined its radiation shielding, mechanical, thermal, electrical, and multifunctional properties. At a diagnostic energy of 40 and 141 keV, the multilayer composites fabricated by a greater molality concentration of 3.2 mmol/g of diverse metallic fillers established extra X-ray shielding capabilities, comprising 0.25 mm Pb-like radiation protection aprons used in the medical sector [13].

Nagaraja et al., investigated the radiation shielding probabilities of commonly used polymers. At energies of 81, 276, 302.8, 356, 383.80, 511, 661.6, 835, 1173, and 1332 keV, the polymer Twaron displayed better absorption ability amid the other commonly used polymers, as well as poly dimethyl among silicon polymers, lead tetragonal coordination polymer among coordination polymers, erbium phosphate hydrate among lanthanide polymers, and polyborazylene among boron polymers [14]. Ambika et al., investigated the electrical, structural, thermal, and gamma attenuation properties of Bi_2_O_3_-filled isophthalic resin-based polymer composites of diverse weight %. The composites were thermally stable, and no mentionable structural changes were detected up to a temperature of 200 °C. Moreover, all the composites showed near to ground conductivity. Peak desertion or peak modification were not observed. The typical crystallite size was 33.03 nm and 37.06 nm considering Scherrer’s formula and W-H plots, respectively. Nevertheless, prepared polymer composites expressed negligible conductivity by adding bismuth oxide. The filled composites holding up to 40 wt% of Bi_2_O_3_ maintained their mechanical strength; however, the mechanical strength declined a little after addition of more Bi_2_O_3_. The linear attenuation coefficient upsurged with the intensification of the filler wt%. The supreme half-value layer was displayed at 2.77 cm [15].

Nagaraja et al., examined silicon polymers with diverse contamination of polymer A-polydimethylsiloxane and other kinds of polymers. The prepared silicon polymer perhydro-polysilaxane showed a greater value for the mass attenuation coefficient and neutron absorption cross-section values than the other studied polymers [16]. Gu et al., investigated basalt fiber containing Er_2_O_3_ particles in the interest of making an innovative radiation shielding compound. The mass attenuation coefficient of the prepared composite was much greater, comprising aluminum in the energy region of 31 to 80 keV. The addition of Er_2_O_3_ particles to the basalt fiber epoxy resin matrix increased the photon energies to 31, 59.5, and 80 keV; however, trivial growth was at 356 and 662 keV [17].

Silicon rubber is considered one of the most flexible polymers. This feature has great applications, especially in the field of medicine. It can be used to protect the body during radiological diagnosis, and therefore it needs to be improved with higher density materials that absorb photons. In this experiment, we prepared silicon rubber (SR) with different concentrations of micro- and nano-sized MgO. We used a HPGe detector to measure radiation attenuation factors at different energies (between 59.6 and 1333 keV). The effect of particle size on attenuation parameters was reported.

## 2. Materials and Methods

A ready-made RTV2 silicone rubber in liquid form was purchased from a local store in Egypt, with its stiffener made in China, in addition to purchasing micro-magnesium oxide from El-Gomhouria Chemicals Company in Cairo, Egypt, with a purity of 97.8% and an average particle size of 60 ± 4 μm. Nano-magnesium oxide was purchased from Nano Tech Company, Egypt, with a purity of 99.8% and an average particle size of 20 ± 5 nm. The nanoparticles were prepared chemically and their purity was confirmed using EDX analysis. Samples were photographed using a transmission electron microscope (TEM) to confirm their size.

The mixing process was manually carried out until it became a single mixture that had no lumps or voids, after which it was poured into molds until dried. The samples were mixed in the same proportions as listed in Table 1, with 2 grams of hardener added to every 50 grams of silicone rubber, then adding either micro- or nano-MgO and mixing well until homogeneous. Then they were poured into molds and left for 24 h.

**Figure 1:**
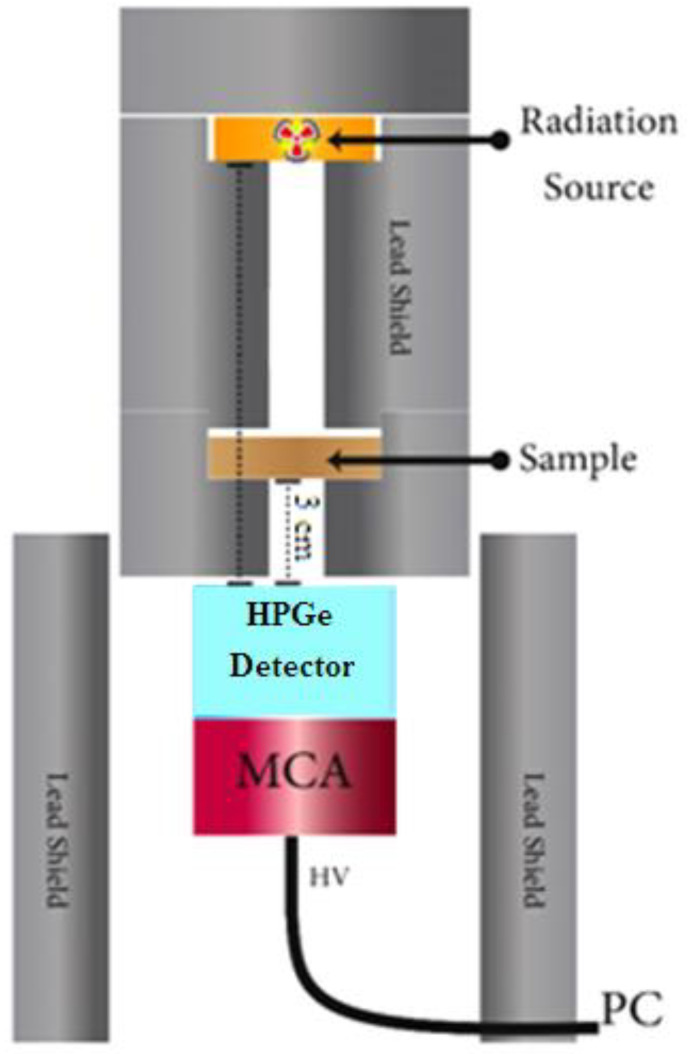
An illustration of the detection measurement.

First, the density was measured using the law of mass per volume; the sample was weighed for mass and the volume was measured by the thickness and the sample radius. Then, a system was designed, as shown in Figure 1, to measure the attenuation coefficient of the existing samples using three radioactive sources (Co-60, Cs-137, and Am-241) and a HPGe detector at the Environmental and Radiation Measurements Laboratory, Institute of Graduate Studies and Research, Alexandria University, Alexandria, Egypt.

The measurement was made in the presence and absence of the absorbed sample to determine the intensity of gamma ray photons in both cases. Genie 2000 software was used to analyze the resulting spectrum and determine the intensity of the photons in the presence of the silicon rubber sample (*N*) and in the absence of the silicon rubber sample (*N*_0_). The linear attenuation coefficient (*LAC*) was experimentally determined using Equation (1) [18,19,20,21,22]:

(1) $$LAC=\frac{1}{d}\;ln\frac{N_{0}}{N}$$

where *d* is the thickness of the sample. From *LAC*, we can determine the half-value layer (*HVL*) by the following Equation (2) [23,24,25,26,27,28,29,30]:

(2) $$HVL=\frac{LN\;\left(2\right)}{LAC}$$

The mean free path (*MFP*) is calculated using Equation (3):

(3) $$MFP=\frac{1}{LAC}$$

The radiation absorption ratio (*RAR*) is an useful quantity for estimating the efficacy of shielding materials and given by Equation (4) [31,32,33,34].

(4) $$RAR\left(\%\right)=\left(1-\frac{N}{N_{0}}\right)\times 100$$

## 3. Results and Discussions

### 3.1. EDX and TEM Results 

**Figure 2:**
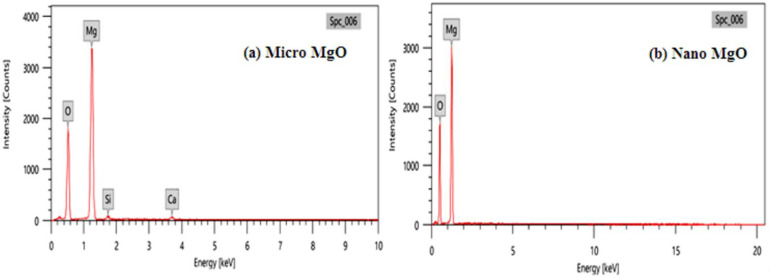
EDX analysis of (**a**) micro-MgO; (**b**) nano-MgO.

**Figure 3:**
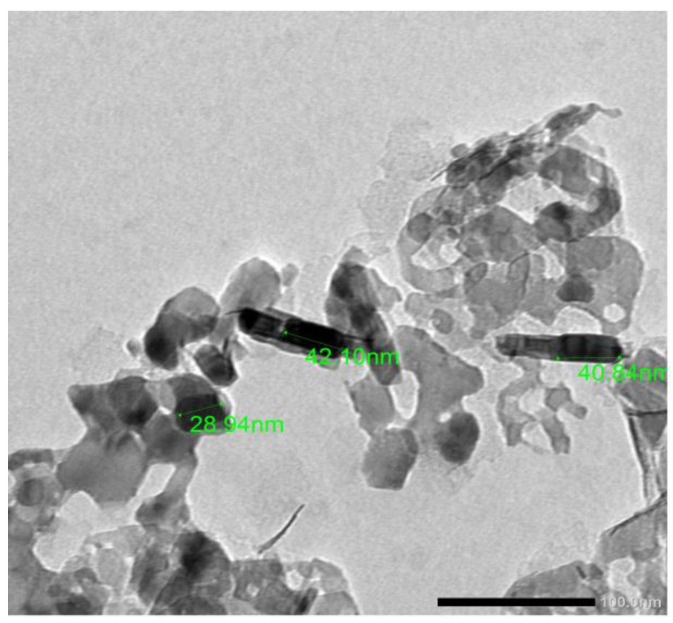
TEM image of nano-MgO.

Before preparing the mixtures, energy dispersive X-ray (EDX) analysis was applied for micro- and nano-MgO powder, as shown in Figure 2. The results indicate that the nano-powder has extremely high purity compared with the micro-powder, where the micro-powder has about 2% impurities. The transmission electron microscope (TEM) was also used, as shown in Figure 3, to ascertain the sizes of the particles. We found that most of the particles had a size of less than 50 nm and an average of 30 nm.

### 3.2. Shielding Results

**Figure 4:**
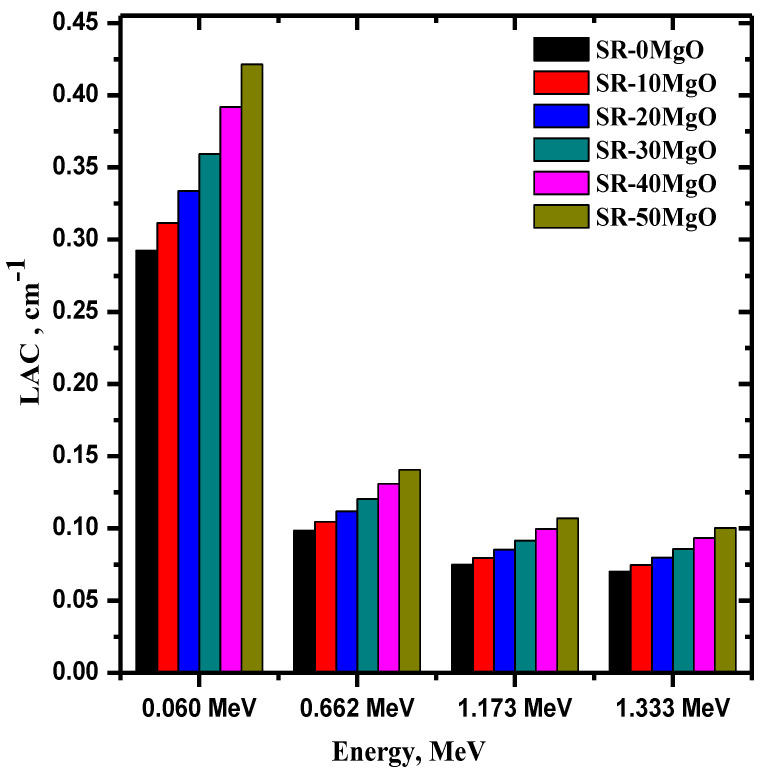
The linear attenuation coefficient of SR-micro-MgO samples as a function of different energies.

The linear attenuation coefficient (LAC) of the five SR-micro-MgO samples (0 to 50% MgO) was plotted as a function of the incoming energy in Figure 4. At all energies, the SR with no MgO had a lower LAC than all the other samples. This observation demonstrates that adding MgO increases the LAC of the system, which is clearly seen in the difference between the LAC values of the samples with 0 and 50% MgO. More specifically, the sample with no MgO has a LAC value of 0.292 cm^−1^ at 0.06 MeV, whereas the SR with 50% MgO has a LAC equal to 0.421 cm^−1^ at the same energy. This result indicates that adding MgO to SR is an effective way to improve its radiation shielding ability. In addition, Figure 4 reveals that the LAC of the SR samples is much higher at 0.06 MeV than at the other energies. For example, for SR-10 MgO, its LAC at 0.06 MeV is equal to 0.311 cm^−1^, which decreases to 0.105 cm^−1^ at 0.662 MeV, to one-third of its previous value. This downward trend is consistent across almost all the other samples. From this, we can observe that SR samples have excellent attenuation ability, no matter the MgO content, at low energies, making it a great candidate for radiation shields designed to absorb low-energy radiation.

**Figure 5:**
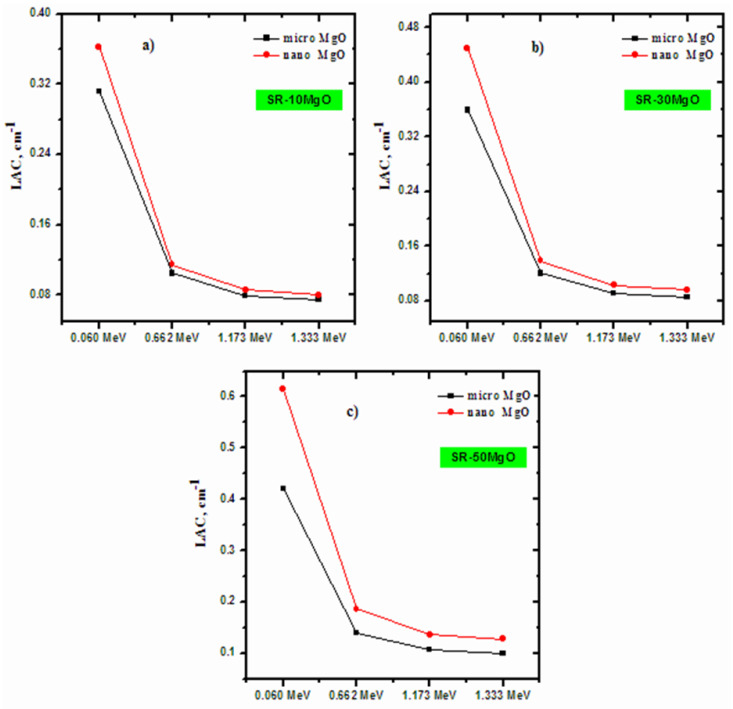
The linear attenuation coefficient of SR with (**a**) 10, (**b**) 30, and (**c**) 50% micro- and nano-MgO samples.

In Figure 5, the LAC for the SR with 10% micro- and nano-MgO (Figure 5a), 30% micro- and nano-MgO (Figure 5b), and 50% micro- and nano-MgO (Figure 5c) were depicted as a function of increasing energy. The aim of Figure 5 is to demonstrate the effect that the size of the MgO particles has on the attenuation ability of the SR samples, and to determine whether micro- or nano-MgO is more effective for radiation shielding purposes. The figure shows that the LAC for SR with nano-MgO is greater than for the micro-MgO samples at all four energies and for all three samples. This means that SR with nano-MgO absorbed more photons than SR with micro-MgO. The SR with nano-MgO contains more MgO particles per gram than SR with micro-MgO. Accordingly, the distribution of nano-sized MgO in the silicon rubber differs from that of micro-sized MgO, resulting in a more uniform dispersion in the silicon rubber. Thus, photons may have a greater chance of interacting with the MgO particles in SR with nano-MgO than in SR with micro-MgO. The difference between the LAC values of the micro- and nano-MgO polymers are greater at lower energies, and the advantage that nanoparticles have over microparticles is not as evident at higher energies. For instance, in Figure 5a, the difference between the LAC values at 0.06 MeV is 16.25%, whereas at 1.333 MeV, it is only 7.12%. This percentage difference was calculated using the following relation:

(5) $$\%\;difference=\frac{LAC_{micro}-LAC_{nano}}{LAC_{micro}}\times 100$$

These same results are found in Figure 5b,c. Additionally, at any single energy, the difference between the LAC values of the micro- and nano-MgO increases when the MgO content is increased in the SR samples. For example, at 0.06 MeV, the difference increases from 16.25% for 10% MgO, to 25.28% at 30% MgO, to 45.82% at 50% MgO, whereas at 1.333 MeV, it increases from 0.080% to 0.086% to 27.95% for the same respective MgO percentages.

**Figure 6:**
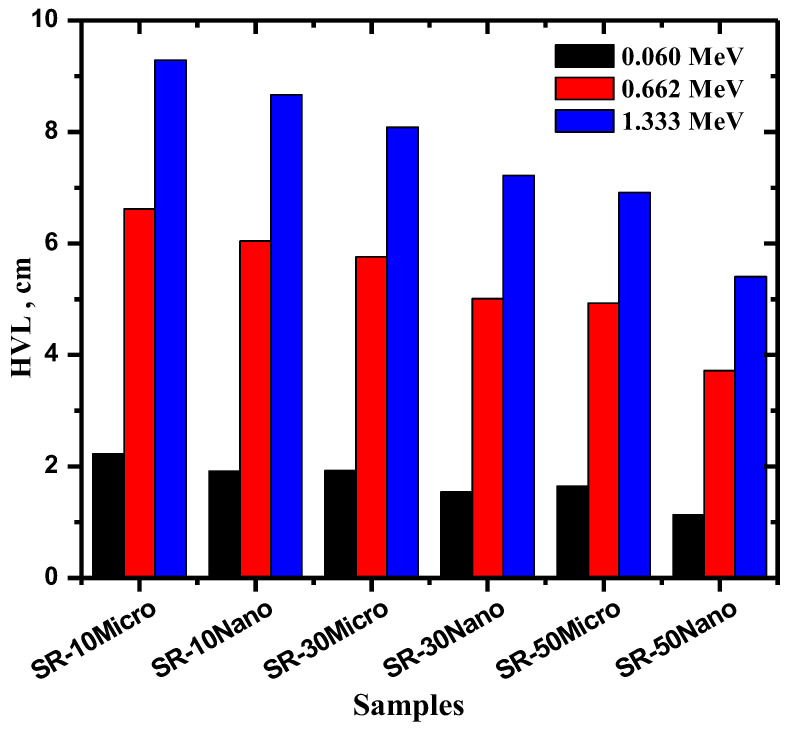
The mean free path (HVL) of SR with 10, 30, and 50% micro- and nano-MgO samples.

The influence of nano- and micro-MgO on the HVL of the SR samples was studied in Figure 6 at different energies. For all four MgO concentrations and particle sizes, the HVL values at 0.06 MeV are very small, at around 2 cm for the samples with 10% MgO and around 1.3 cm for those with 50% MgO. Increasing the energy of the incoming particles causes a notable increase in the HVL values for all MgO concentrations, where the maximum HVL is found in samples with 10% MgO. When comparing the particle sizes of the samples, we found that the HVL values for nano-MgO were lower than those for micro-MgO. For example, at 10% micro- and nano-MgO, the HVL values were equal to 2.225 and 1.914 cm at 0.06 MeV, respectively, and equal to 6.62 and 6.04 cm at 0.662 MeV, respectively. When looking closer at the samples with 50% MgO, the results show that the HVL values decrease from 4.93 to 3.72 cm at 0.662 MeV if micro-MgO is replaced by nano-MgO, and from 6.916 to 5.405 cm at 1.333 MeV for micro- and nano-MgO, respectively. These results demonstrate the usefulness of using nano-MgO when developing new and enhanced radiation shielding materials.

**Figure 7:**
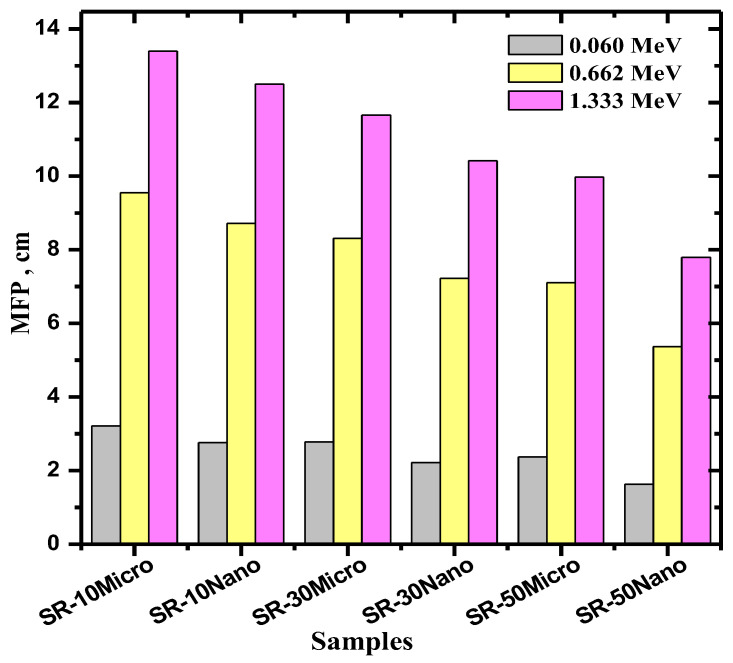
The mean free path (MFP) of SR with 10, 30, and 50% micro- and nano-MgO samples.

Figure 7 illustrates the mean free path (MFP) of the samples at three chosen energies [35,36,37]. The MFP values show that the SR polymers with nano-MgO have a lower MFP than those with micro-MgO. For example, for 30% MgO, SR with micro-MgO has an MFP of 2.784 cm at 0.060 MeV, 8.313 cm at 0.662 MeV, and 11.661 cm at 1.333 MeV, whereas SR with nano-MgO of the same percentage has MFP values equal to 2.222, 7.224, and 10.415 cm for the same respective energies. This downward trend reinforces the conclusion that nano-MgO particles are more effective at absorbing radiation than micro-MgO particles, for all tested energies and concentrations. Furthermore, the MFP values decrease with increasing MgO concentrations. At 0.662 MeV, the MFP values are equal to 9.550, 8.313, and 7.112 cm for 10, 30, and 50% micro-MgO, respectively, whereas they are equal to 8.715, 7.224, and 5.367 cm for 10, 30, and 50% nano-MgO, respectively. These results demonstrate that increasing the MgO concentration improves the shielding ability of SR.

**Figure 8:**
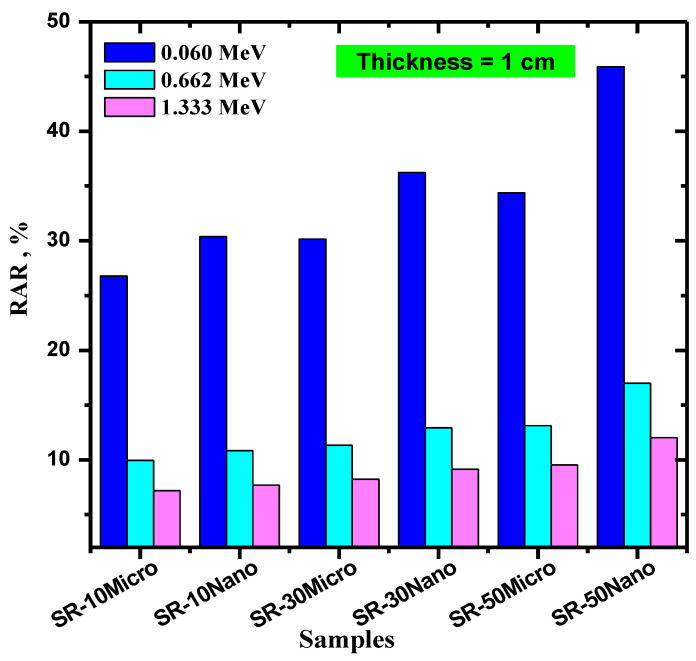
The radiation absorption ratio (RAR) of a 1 cm thick SR with 10, 30, and 50% micro- and nano-MgO samples.

The radiation absorption ratio (RAR) of 1 cm thick SR samples were calculated and graphed in Figure 8 at several energies. At any single energy, the RAR for SR with nano-MgO is higher than that of SR with micro-MgO, which means that the nano-MgO SR absorbs more photons than its microparticle counterpart. For example, the RAR values for 10% micro-MgO are 26.76, 9.94, and 7.19% at 0.060, 0.662, and 1.333 MeV, respectively, whereas for 10% nano-MgO they are equal to 30.38, 10.84, and 7.68%, for the same respective energies. Moreover, RAR increases as more MgO is added to the SR, meaning that an effective way to enhance the radiation absorption ability of the SR samples is to use high amounts of MgO nanoparticles. For instance, at 0.662 MeV, the RAR values increase from 9.94% for 10% micro-MgO, to 11.33% for 30% micro-MgO, to 13.12% for 50% micro-MgO. Meanwhile, for nano-MgO at the same energy, they are equal to 10.84, 12.93, and 17.00% for the same respective concentrations.

**Figure 9:**
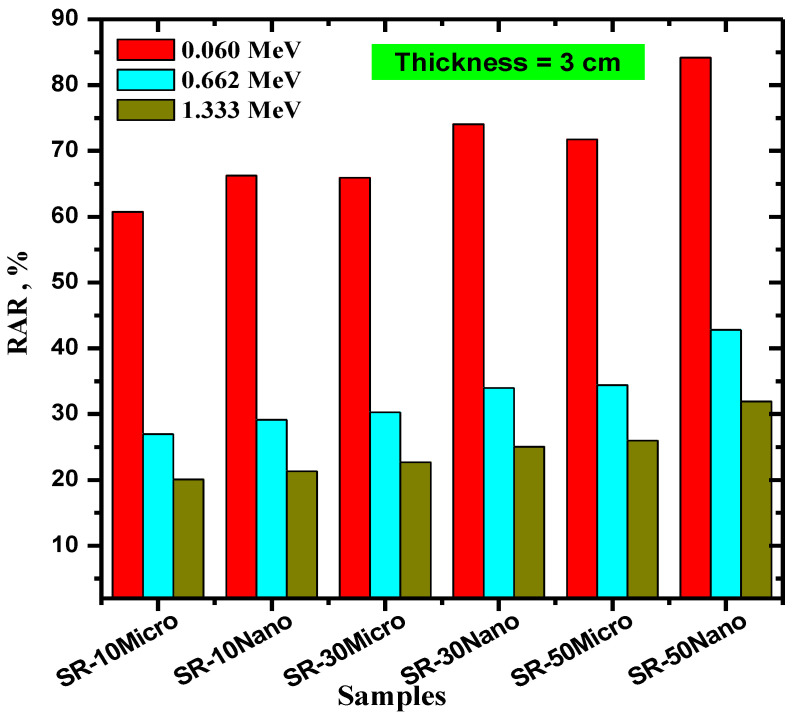
The radiation absorption ratio (RAR) of a 3 cm thick SR with 10, 30, and 50% micro- and nano-MgO samples.

The purpose of Figure 9 is to analyze the influence of the thickness of the SR samples on their photon absorption ability. The RAR values were once again calculated, but for samples that were 3 cm thick instead of 1 cm. The figure reveals that the RAR of all the polymers increases as thickness increases. For instance, at 0.06 MeV, the RAR value of SR with 10% micro-MgO increases from 26.76 to 60.72% as its thickness increases from 1 to 3 cm, whereas the RAR values of SR with 50% micro-MgO at the same energy increase from 34.39 to 71.75% as this thickness increases. This trend demonstrates that if the space is available, increasing the thickness of the SR shields to 3 cm has a significant improvement on its shielding performance.

## 4. Conclusions

We prepared silicon rubber (SR) with different concentrations of micro- and nano-MgO. Using a HPGe detector, we successfully measured the LAC and studied the influence of MgO particle size on this and other parameters. The results revealed that the LAC values for SR with nano-MgO are higher than those with micro-MgO. The difference between the LAC values of the micro- and nano-MgO polymers were greater at lower energies, and the advantage that nanoparticles have over microparticles was not as evident at higher energies. The MFP for both SRs with MgO (in either micro- and nano-sizes) had a lower MFP than pure SR, which reaffirmed that adding MgO to SR is an effective way to improve its radiation shielding ability. From the HVL results, a certain thickness of SR with nano-MgO is required to reduce the intensity of the low-energy photons. From the RAR results, we concluded that if the space is available, increasing the thickness of the SR shields to 3 cm has a significant improvement on its shielding performance. Based on our results, we can conclude that prepared polymers have potential to be used as protection in different medical applications where low energy photons are utilized.

## Figures and Tables

**Table 1:** Codes, weight fraction, and densities of SR-MgO composites.

Codes	Compositions (wt%)			Density (g × cm^−3^)
	SR	MgO		
		Micro	Nano	
SR-0MgO	100	-		1.180 ± 0.008
SR-10mMgO	90	10	-	1.264 ± 0.007
SR-10nMgO	90	-	10	1.268 ± 0.011
SR-20mMgO	80	20	-	1.361 ± 0.009
SR-30mMgO	70	30	-	1.469 ± 0.006
SR-30nMgO	70	-	30	1.479 ± 0.006
SR-40mMgO	60	40	-	1.621 ± 0.007
SR-50mMgO	50	50	-	1.754 ± 0.008
SR-50nMgO	50	-	50	1.760 ± 0.004

## Data Availability

The data presented in this study are available on request from the corresponding author.
